# Fatty Acids and Their Roles in Cardiac Physiology and Pathology: Mechanistic and Interventional Studies

**DOI:** 10.3390/nu18091429

**Published:** 2026-04-30

**Authors:** Rahul Mallick, Prasenjit Bhowmik, Premanjali Chowdhury, Asim K. Duttaroy

**Affiliations:** 1 A.I. Virtanen Institute for Molecular Sciences, Faculty of Health Sciences, University of Eastern Finland, FI-70211 Kuopio, Finland; rahul.mallick@uef.fi; 2 Department of Environmental, Biological and Pharmaceutical Sciences and Technologies (DiSTABiF), University of Campania “Luigi Vanvitelli”, 81100 Caserta, Italy; prasenjit.bhowmik@studenti.unicampania.it; 3 Institute of Biostructures and Bioimaging, Consiglio Nazionale delle Ricerche (IBB-CNR), Via Pietro Castellino, 111, 80131 Naples, Italy; 4 Institute of Public Health and Clinical Nutrition, School of Medicine, Faculty of Health Sciences, University of Eastern Finland, FI-70211 Kuopio, Finland; premacho@student.uef.fi; 5 Department of Nutrition, Institute of Medical Sciences, Faculty of Medicine, University of Oslo, 0316 Oslo, Norway

**Keywords:** fatty acids, cardiac physiology, cardiovascular diseases, lipotoxicity, *n*-3 fatty acids, trans fats, dietary interventions, cardiac metabolism

## Abstract

Fatty acids serve dual roles in cardiac physiology: as energy substrates and as precursors of bioactive lipid mediators (prostaglandins, leukotrienes, oxylipins) from *n*-3/*n*-6 PUFAs that regulate inflammation, thrombosis, and remodeling. Saturated, monounsaturated, and trans fatty acids modulate metabolism and membrane function, thereby shaping these pathways. Clinically, *n*-3 long-chain PUFAs (EPA and DHA) reduce cardiovascular mortality and aid postischemic remodeling; however, high doses increase the risk of atrial fibrillation. By contrast, trans and saturated fatty acids promote dyslipidemia, dysfunction, and higher rates of coronary artery disease and heart failure. Mechanistically, fatty acid uptake via FABPpm, CD36 (FAT), and FATPs, along with β-oxidation and PPAR signaling, regulates metabolism, while COX/LOX/CYP pathways generate eicosanoids and resolvins that influence inflammation and repair. This review synthesizes evidence on the roles of fatty acids and oxylipins in lipotoxicity, heart failure, ischemia–reperfusion, and arrhythmias, and evaluates dietary and supplemental interventions to optimize cardiac lipid metabolism, aligning with fatty acid signaling.

## 1. Introduction

Fatty acids play a crucial role in cardiac physiology, serving as essential energy substrates and as precursors to bioactive lipid mediators that regulate myocardial function [1]. Among these, the essential *n*-3 and *n*-6 polyunsaturated fatty acids (PUFAs) are of particular interest because they give rise to prostaglandins, leukotrienes, and a broad oxylipin network that modulates vascular tone, inflammation, thrombosis, and tissue remodeling in the heart [1]. Saturated, monounsaturated, trans, and essential PUFAs each shape cardiomyocyte metabolism and membrane composition, thereby activating distinct mediator pathways in health and disease [2,3].

Different fatty acids have opposing clinical effects. *n*-3 long-chain PUFAs such as eicosapentaenoic acid (EPA, 20:5*n*-3) and docosahexaenoic acid (DHA, 22:6*n*-3) can lower overall cardiovascular mortality. However, high-dose pharmaceutical formulations (≥4 g/day of EPA/DHA ethyl esters) used in secondary prevention trials (REDUCE-IT, STRENGTH, VITAL-Rhythm) show a modest increase in the risk of atrial fibrillation (odds ratio 1.2–1.6) among patients with established cardiovascular disease or diabetes. By contrast, diets rich in industrial trans fatty acids (TFAs) and excessive long-chain saturated fatty acids (SFAs) promote dyslipidemia, endothelial dysfunction, and proarrhythmic remodeling, increasing the incidence of coronary artery disease, heart failure, and sudden cardiac death [4,5,6]. These divergent clinical outcomes reflect not only bulk lipid levels but also distinct patterns of downstream eicosanoids and specialized pro-resolving mediators derived from arachidonic acid (AA, 20:4*n*-6), EPA, and DHA.

At the molecular level, cardiac NEFA uptake occurs through coordinated transport and metabolic systems. Uptake into cardiomyocytes is mediated by transporters such as CD36/FAT (fatty acid translocase), plasma membrane fatty acid-binding protein (FABPpm), and fatty acid transport proteins (FATPs). Once inside, fatty acids are activated by acyl-CoA synthetases and imported into mitochondria for β-oxidation [7,8]. In the healthy adult myocardium, under fasting or moderate-workload conditions, approximately 60–70% of ATP production derives from fatty acid β-oxidation, although this contribution declines during fed states (when insulin promotes a preference for glucose) or in heart failure (when ketone and lactate utilization increase). Beyond serving as fuels, fatty acids and their oxylipin metabolites act as ligands for nuclear receptors such as peroxisome proliferator-activated receptors (PPARs), thereby regulating transcriptional programs that govern substrate preference, mitochondrial biogenesis, oxidative stress responses, and inflammatory tone. Conversely, chronic overload of long-chain fatty acids—particularly in obesity and diabetes—can precipitate lipotoxic cardiomyopathy, characterized by the accumulation of toxic lipid intermediates, mitochondrial dysfunction, and activation of inflammatory and apoptotic signaling pathways.

Given this dualistic nature, a mechanistic understanding of how fatty acids—especially essential *n*-3 and *n*-6 series—are converted into prostaglandins, leukotrienes, and related oxylipins, and how these mediators influence cardiac structure and function, is critical for developing targeted dietary and pharmacological interventions. This review follows a pathway from essential fatty acids to lipid mediators to cardiac outcomes, while also considering how different fatty acids and overall dietary patterns influence this axis. In this context, we synthesize current evidence on: (1) the physiological roles of distinct fatty acid species in myocardial energy production and lipid mediator signaling; (2) the molecular mechanisms linking fatty acid and oxylipin dysregulation to lipotoxicity, inflammation, oxidative stress, and arrhythmogenesis; (3) clinical and epidemiological data relating dietary fatty acid profiles and EFA-derived mediators to cardiovascular outcomes; and (4) interventional strategies—including dietary modifications, nutraceutical supplementation, and molecular therapies—aimed at optimizing cardiac fatty acid and oxylipin metabolism. This review adopts a narrative approach because the topic spans multiple dimensions that cannot be addressed by a narrowly defined research question. We provide a narrative overview of representative experimental and clinical literature on fatty acids and cardiac outcomes, emphasizing mechanistic and translational findings rather than an exhaustive systematic search.

## 2. Types of Fatty Acids: Structure, Sources, and Cardiac Relevance

**Figure 1:**
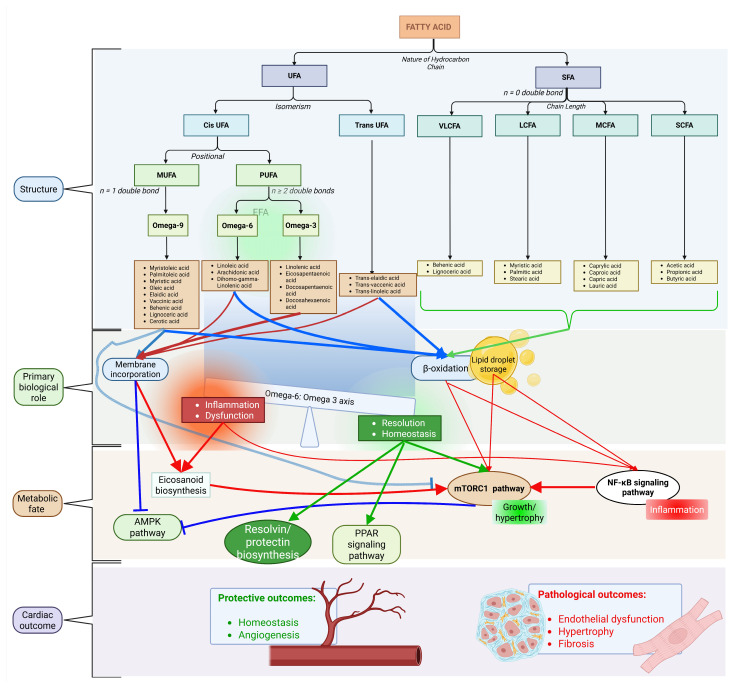
Overview of fatty acid classification, metabolic pathways, and their roles in cardiovascular regulation. Fatty acids are classified based on chain length and degree of saturation into saturated (SFAs), monounsaturated (MUFAs), and polyunsaturated fatty acids (PUFAs). Upon cellular uptake, fatty acids are directed toward β-oxidation for energy production, storage in lipid pools, or incorporation into membrane phospholipids. Polyunsaturated fatty acids, particularly *n*-3 and *n*-6 species, serve as precursors for bioactive lipid mediators generated through COX, LOX, and CYP pathways. These metabolic and signaling processes collectively regulate key aspects of cardiac physiology, including inflammation, vascular tone, and structural remodeling. Imbalances in fatty acid composition, such as excess SFAs or altered *n*-6/*n*-3 ratios, contribute to pathological outcomes, including lipotoxicity, endothelial dysfunction, and fibrosis. This figure was created based on findings discussed in the text [9,10,11,12,21,22,23].

The cardiometabolic effects of fatty acids depend on three key structural features: chain length, saturation, and double-bond geometry. These features determine not only β-oxidation efficiency but also the availability of fatty acids as substrates for phospholipase A_2_, which releases them from membrane phospholipids for subsequent enzymatic conversion via cyclooxygenase (COX), lipoxygenase (LOX), and cytochrome P450 (CYP) pathways into prostaglandins, leukotrienes, and specialized pro-resolving mediators (SPMs) (Figure 1) [9]. This structure–function relationship is particularly important for essential fatty acid biology. The essential *n*-3 and *n*-6 PUFAs—namely linoleic acid (LA, 18:2*n*-6) and α-linolenic acid (ALA, 18:3*n*-3)—cannot be synthesized de novo. Instead, they serve as indispensable dietary precursors that are metabolized to AA, EPA, and DHA. These long-chain PUFAs subsequently generate prostaglandins, thromboxanes, leukotrienes, lipoxins, and SPMs, all of which regulate cardiac inflammation, vascular tone, thrombosis, and remodeling. By contrast, non-essential SFAs, monounsaturated fatty acids (MUFAs), and TFAs lack these enzymatic conversion pathways. However, they modulate myocardial pools of essential fatty acids (EFAs), influence membrane microdomain organization, and contribute to lipotoxic stress, thereby indirectly shaping oxylipin biosynthesis and signaling.

SFAs, which lack double bonds, are not a homogeneous class with respect to cardiovascular risk. Palmitic acid (16:0), abundant in palm oil and animal fats, is frequently associated with adverse outcomes. It accumulates as ceramides and diacylglycerols, which drive insulin resistance, endoplasmic reticulum stress, and atherogenesis through TLR4 and NFκB activation [10,11,12]. Excess SFA exposure also alters phospholipid remodeling, reducing membrane incorporation of *n*-6 and *n*-3 EFAs and promoting a pro-inflammatory oxylipin profile [13,14,15,16]. Palmitic acid is actively metabolized via desaturation (to palmitoleic acid, 16:1*n*-7) and elongation (to stearic acid, 18:0), serving as a crucial precursor for MUFA synthesis through stearoyl-CoA desaturase 1 (SCD1). By contrast, stearic acid (18:0) is rapidly desaturated by SCD1 into oleic acid (OA, 18:1*n*-9), which minimizes ceramide formation and exerts neutral effects on LDL cholesterol [17,18]. Very-long-chain SFAs (VLSFAs; here defined as C20–C24, though nomenclature varies) enrich cardiac sphingolipids and may influence lipid raft organization of oxylipin receptors, though direct cardiac data remain limited [9].

Unlike EFAs, MUFAs do not produce oxylipins directly but indirectly regulate LA and ALA metabolism by competing for elongase enzymes (ELOVL5/6) and through sterol regulatory element-binding protein 1c (SREBP-1c)-mediated suppression of fatty acid desaturase 1 and 2 (FADS1/2) expression, thereby optimizing tissue *n*-6/*n*-3 ratios and limiting AA availability for pro-inflammatory series-2 prostaglandins [19,20]. OA also stabilizes mitochondrial membranes and enhances PPARα-driven fatty acid oxidation, indirectly supporting the metabolic milieu for EFA-derived mediator production [20].

The *n*-6 PUFA LA, found abundantly in seed oils, is elongated and desaturated to AA. AA serves as the canonical substrate for two major pathways: (1) COX-2-derived PGE_2_ and TXB_2_, which are vasoconstrictive and pro-thrombotic; and (2) 5-LOX-derived LTB_4_ and LTC_4_, which are chemoattractants and neutrophil activators [24,25]. Excess dietary *n*-6 relative to *n*-3 (>10:1 in Western diets) promotes AA dominance and myocardial inflammation post-ischemia [21].

By contrast, the *n*-3 PUFA ALA, found in flaxseed and walnuts, is converted inefficiently (approximately 5–10%) to EPA and DHA. Marine sources provide preformed EPA and DHA, bypassing this inefficient conversion. These *n*-3 PUFAs yield less inflammatory series-3 prostanoids (PGE_3_ and TXB_3_), as well as 5-LOX-derived resolvins, protectins, and maresins, and CYP-derived epoxyeicosatrienoic acids (EETs), all of which functionally oppose pro-inflammatory AA-derived products [26]. EPA and DHA incorporation into the sarcolemma alters lipid rafts, modulates ion channel function (sodium and calcium channels), and activates GPR120 signaling to suppress NFκB while promoting inflammation resolution; optimal cardiac benefits occur at tissue *n*-6/*n*-3 ratios below 4:1 [27]. Clinical trials show that high-dose EPA and DHA reduce cardiovascular mortality but raise atrial fibrillation risk, likely through electrophysiological remodeling [27].

Industrial TFAs, such as elaidic acid (18:1*n*-9t) from partial hydrogenation, are non-essential and uniquely atherogenic. They elevate the LDL to high-density lipoprotein (HDL) cholesterol ratio while promoting endothelial dysfunction. TFAs disrupt membrane order, potentially mislocalizing COX and LOX enzymes or G protein-coupled receptor (GPCR) oxylipin receptors within lipid rafts, though direct data on cardiac mediators remain sparse; global bans on industrial TFAs reflect their consistent CVD hazard, independent of EFA pathways [28,29].

Dietary fatty acids enter circulation via chylomicrons and very low-density lipoproteins (VLDL), undergo lipoprotein lipase (LPL)-mediated hydrolysis, and enter cardiomyocytes via CD36/FAT and fatty acid transport proteins (FATPs) to fuel β-oxidation (providing approximately 60–70% of myocardial ATP) and to establish EFA pools for phospholipase A_2_-mediated release and subsequent COX/LOX/CYP conversion into oxylipins [9]. Thus, dietary composition directly shapes not only energy homeostasis but also the myocardial substrate landscape for prostaglandin, leukotriene, and SPM biosynthesis, which governs cardiac health and disease.

In summary, the cardiovascular impact of dietary fatty acids cannot be predicted from saturation alone. Chain length (VLSFAs protective vs. longer-chain SFAs harmful), double-bond geometry (cis vs. trans), and the *n*-6/*n*-3 ratio collectively determine whether fatty acids promote inflammation and thrombosis or support resolution and cardiac protection. This mechanistic framework underpins the subsequent sections on cardiac physiology and pathology.

## 3. Fatty Acids in Cardiac Physiology

Fatty acids sustain cardiac contractile function through two distinct roles: (1) as primary energy substrates, providing approximately 60–70% of myocardial ATP via β-oxidation; and (2) as precursors for oxylipins that modulate excitation–contraction coupling, inflammation, and bioenergetics [1,30,31]. This section examines how essential *n*-3 and *n*-6 PUFAs—along with other fatty acid classes—support physiological cardiac work. We emphasize how membrane incorporation of these fatty acids, followed by phospholipase A_2_-mediated release, establishes substrate pools for COX-, LOX-, and CYP-derived prostaglandins, leukotrienes, and SPMs. Non-essential fatty acids provide the metabolic context by influencing both the incorporation of EFAs into sarcolemmal and phospholipid membranes and the function of receptor signaling domains.

### 3.1. Role of Fatty Acids in Cardiac Metabolism

The myocardium exhibits metabolic flexibility but preferentially oxidizes long-chain fatty acids (LCFAs such as palmitate) for efficient ATP production during fasting or moderate workloads. Oxidative metabolism generates approximately 95% of cardiac energy, with fatty acid β-oxidation predominating over glycolysis even in heart failure, where utilization of ketones and lactate increases [30]. Essential PUFAs contribute minimally to bulk ATP (approximately 1–5% of the oxidation rate) but are critical for maintaining cardiolipin composition and mitochondrial cristae structure. Their sn-2 phospholipid esterification enables rapid phospholipase A_2_-mediated release for oxylipin biosynthesis under stress [9].

### 3.2. Fatty Acids as Energy Sources for the Heart

**Figure 2:**
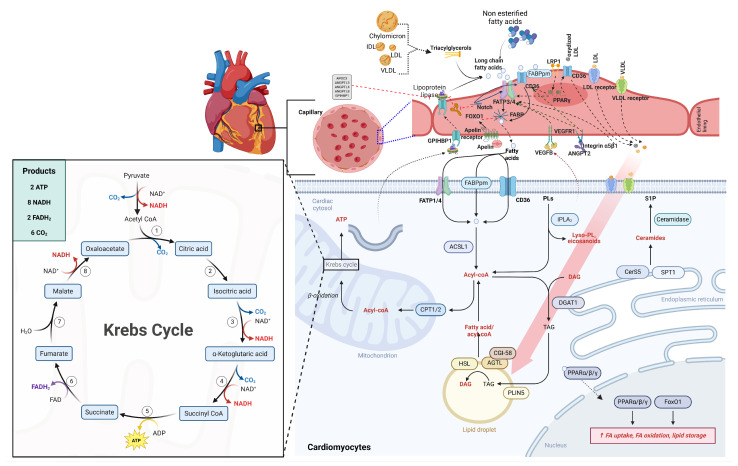
Fatty acid uptake, intracellular trafficking, and metabolic fate in cardiomyocytes. Circulating triglycerides in chylomicrons and VLDL are hydrolyzed by lipoprotein lipase (LPL) at the coronary endothelium, releasing non-esterified fatty acids (NEFAs). These fatty acids enter cardiomyocytes primarily via CD36 and fatty acid transport proteins (FATPs), with additional transfer facilitated by membrane-associated binding proteins. Intracellularly, fatty acids are converted to acyl-CoA and directed toward mitochondrial β-oxidation for ATP production or stored as triglycerides in lipid droplets. Mitochondrial uptake is regulated by carnitine palmitoyltransferase 1 (CPT1), a key rate-limiting step. This coordinated system links fatty acid availability to energy production and lipid signaling in the heart. Figure 2 was drawn based on findings discussed in the text [7,9,22,30,32,33,34].

Dietary lipids circulate as chylomicron and VLDL triglycerides, undergo hydrolysis by endothelial LPL, and deliver non-esterified fatty acids (NEFAs) or free fatty acids (FFAs) bound to albumin for cardiac uptake (Figure 2). During insulin suppression (fasting/exercise), adipocyte hormone-sensitive lipase (HSL) releases additional NEFAs, whereas insulin normally inhibits this flux to favor glucose [22]. β-oxidation of long-chain fatty acids (LCFAs) yields ~2.8× more ATP per mole of fatty acid than glucose oxidation but is ~12% less oxygen-efficient (106 ATP/O_2_ for palmitate vs. 120 ATP/O_2_ for glucose), explaining myocardial LCFA preference during oxygen-abundant conditions despite the lower P/O ratio. However, *n*-3 PUFAs like DHA resist complete oxidation and instead enrich mitochondrial membranes, thereby optimizing the efficiency of the electron transport chain [9,23].

### 3.3. Mechanisms of Fatty Acid Uptake and Oxidation in Cardiac Cells

Cardiac NEFA uptake occurs via CD36 and FATPs in coronary endothelial cells, and trans-endothelial transfer to cardiomyocytes is facilitated by plasma membrane fatty acid-binding protein (FABPpm) (Figure 2). Fatty acid translocase (FAT)/CD36, a PPAR-regulated protein responsible for 60–80% of LCFA flux, shows unique cardiac upregulation during fasting, unlike in skeletal muscle or adipose tissue [8,30,32,33,34]. Cytosolic fatty acid-binding proteins (FABPs) shuttle acyl-CoA esters to mitochondria, where carnitine palmitoyltransferase 1 (CPT1) converts them to acylcarnitines for β-oxidation. This rate-limiting step is inhibited by malonyl-CoA (an acetyl-CoA carboxylase (ACC) product) [8]. LPL deficiency impairs triglyceride uptake but spares basal function via NEFA compensation; stress-exposed LPL-null hearts fail due to ATP substrate limitation [33].

PPARα (abundant in cardiomyocytes) transcriptionally coordinates this system by activating CD36, FABPs, CPT1, and medium-chain acyl-CoA dehydrogenase (MCAD), and Kruppel-like factor 15 (KLF15) enhances PPARα expression [35,36,37,38]. During energy stress, AMP-activated protein kinase (AMPK) promotes fatty acid oxidation by phosphorylating ACC, thereby relieving the inhibition of CPT1 [35]. Essential *n*-3 PUFAs uniquely activate PPARα and PPARδ and free fatty acid receptor 4 (GPR120/FFAR4) to enhance fatty acid oxidation capacity, while their oxylipin metabolites (e.g., CYP2J-derived epoxy-fatty acids) vasodilate coronary arteries, thereby matching substrate delivery to demand [39].

### 3.4. Impact of Different Types of Fatty Acids on Cardiac Function

**Figure 3:**
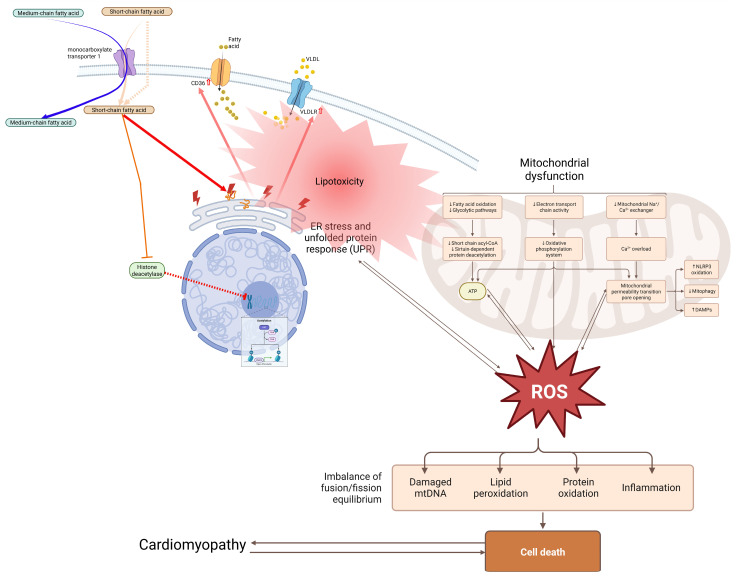
Mechanisms of fatty acid-induced lipotoxicity and mitochondrial dysfunction in cardiomyocytes. Excess fatty acid uptake via transporters such as CD36 and lipoprotein-derived pathways leads to intracellular lipid accumulation. Surplus fatty acids are diverted into toxic intermediates, including ceramides and diacylglycerols, which trigger endoplasmic reticulum stress and inflammatory signaling. Mitochondrial function is impaired, resulting in reduced β-oxidation efficiency, decreased ATP production, and increased reactive oxygen species (ROS) generation. These alterations disrupt calcium handling, promote oxidative damage, and activate cell death pathways, ultimately contributing to cardiomyocyte dysfunction and the development of cardiomyopathy. Figure 3 was created based on lipotoxicity, ER stress, and mitochondrial failure as discussed in [30,40,41,42,43,44,55].

Under physiological conditions, distinct fatty acids play specialized roles beyond bulk energetics (Figure 3):

SFAs: palmitate and stearate provide efficient ATP, but excess induces ceramide-driven lipotoxicity; stearate’s rapid conversion to OA by SCD1 minimizes this risk [40,41,42,43,44].

MUFAs: OA suppresses the NLR family pyrin domain containing 3 (NLRP3) inflammasome, stabilizes mitochondria, and competes with *n*-6 fatty acids for desaturases to optimize the AA/EPA balance [45,46].

*n*-3 PUFAs: EPA/DHA incorporate into the sarcolemma (altering Na^+^/Ca^2+^ channel kinetics and reducing excitability), activate GPR120/NFκB suppression, generate resolvins/protectins via 5-LOX, and stabilize cardiolipin to prevent mtROS—effects that explain anti-arrhythmic and anti-fibrotic actions [31,47,48,49].

*n*-6 PUFAs: AA sustains baseline prostanoid production (PGE_2_ for vascular tone), but excess favors LTB_4_-driven inflammation when the *n*-6/*n*-3 ratio >10:1 [9].

TFAs: have a minimal physiological role; they disrupt membrane order and impair ion channel function in myocytes.

Treatment with DHA can alter the expression of microRNAs (miRNAs) involved in lipid metabolism and angiogenesis in cardiomyocytes (e.g., miR-107 and miR-223), whereas saturated fat consumption deregulates miRNAs implicated in cardiac hypertrophy and fibrosis [50,51].

Free fatty acid receptors (FFARs) exhibit chain-length specificity. In cardiomyocytes, GPR120 mediates NFκB suppression by *n*-3 PUFAs via β-arrestin signaling [52,53]. GPR120 is a medium- and long-chain fatty acid-sensing GPCR that has also been identified alongside FFAR1 (GPR40) [54].

These baseline functions establish myocardial resilience, whereas pathological EFA and oxylipin shifts (explored next) precipitate dysfunction. Membrane *n*-3 enrichment particularly lowers the resting potential and action potential duration through direct channel modulation, reducing susceptibility to arrhythmias independent of metabolism [49].

In summary, the healthy heart derives most of its ATP from fatty acid β-oxidation, but different fatty acid classes serve distinct non-energetic functions: *n*-3 PUFAs provide anti-arrhythmic and anti-inflammatory signaling, *n*-6 PUFAs sustain baseline prostanoid tone, MUFAs offer metabolic flexibility, and excess SFAs (particularly palmitate) create lipotoxic risk. This functional specialization explains why dietary fatty acid composition—not just total fat intake—determines cardiac health outcomes.

## 4. Molecular Pathways and Emerging Research in Fatty Acid–Cardiac Interactions

Fatty acids regulate cardiac physiology through transcriptional, receptor-mediated, inflammatory, and mitochondrial pathways. Essential *n*-3 and *n*-6 PUFAs serve as substrates for COX/LOX/CYP-derived prostaglandins (PGs), leukotrienes (LTs), and SPMs that fine-tune excitation–contraction coupling, bioenergetics, and stress responses [9]. This section examines these pathways with an explicit focus on oxylipin biosynthesis and signaling, while the effects of SFAs, MUFAs, and TFAs are contextualized as modulators of EFA pools and membrane microdomains. Each pathway is linked to cardiac functional outcomes, including arrhythmia, contractility, and fibrosis.

### 4.1. Transcriptional and Nuclear Receptor Pathways

Fatty acids modulate cardiac physiology through distinct molecular pathways, with SFAs and UFAs exerting differential effects on key signaling cascades.

#### 4.1.1. PPAR Regulation

Peroxisome proliferator-activated receptors (PPARs), a subfamily of nuclear hormone receptors, are ligand-activated transcription factors that regulate diverse biological functions [56]. The three PPAR isoforms, alpha (α), beta (β)/delta (δ), and gamma (γ), show differential expression across tissues [57]. PPARα and PPARγ primarily regulate glucose homeostasis, insulin sensitivity, and lipid metabolism; their agonists are used to treat hyperlipidemia and type 2 diabetes. By contrast, PPARβ/δ helps regulate FAO, glucose homeostasis, lipid metabolism, and inflammation. Its agonists are used to treat cardiovascular and metabolic disorders [56].

Fatty acids serve as endogenous ligands for PPARs, modulating gene expression related to fatty acid oxidation (e.g., CPT1, FABPs, and acyl-CoA oxidase 1 (ACOX1)), lipid transport, and glucose handling [58,59,60,61,62,63]. PPARs translocate to the nucleus, bind ligands, dimerize with the retinoid X receptor (RXR), and then bind DNA regulatory regions upstream of target genes [64]. Notably, PPARα regulates cholesterol synthesis via sterol regulatory element-binding protein 2 (SREBP-2) and suppresses inflammatory gene expression by antagonizing NFκB [65]. *n*-3 PUFAs (EPA and DHA) are potent PPARα and PPARγ ligands, and their COX-derived metabolite 15-deoxy-Δ12,14-prostaglandin J_2_ (15d-PGJ_2_) further activates PPARγ-mediated anti-inflammatory signaling in cardiomyocytes [31,65]. PPAR transcription is regulated by Kruppel-like factors (KLFs): KLF5 binds the PPARα promoter, KLF4 regulates mitochondrial biogenesis, and KLF15 associates with PPARα [36].

#### 4.1.2. AMP-Activated Protein Kinase (AMPK) Energy Sensing

As a cellular energy sensor, AMPK is traditionally activated by a decline in energy status, indicated by increases in the AMP:ATP and ADP:ATP ratios. After activation, AMPK promotes ATP-producing catabolic pathways and inhibits energy-consuming processes to restore energy homeostasis [66]. It phosphorylates ACC, relieving inhibition of CPT1, thereby decreasing fatty acid synthesis and enhancing FAO. FAO, in turn, activates SREBP-1c to suppress de novo lipogenesis [67,68].

FFAs can act as both activators and inhibitors of AMPK. For instance, nitrated OA can activate AMPK in endothelial cells, whereas increased intake of industrial foods and frequent meals appears to increase SFAs, which inactivate AMPK [54,69]. In cardiomyocytes, *n*-6 fatty acid-derived LTB_4_ inhibits AMPK via p38 mitogen-activated protein kinase (p38MAPK), whereas *n*-3-derived EETs activate it, protecting against ischemia–reperfusion injury [31,70].

#### 4.1.3. Sirtuin Modulation

Sirtuins (SIRTs) are highly conserved nicotinamide adenine dinucleotide (NAD^+^)-dependent deacetylases and ADP-ribosyl transferases. Seven isoforms (SIRT1–SIRT7) have been identified in mammals [71,72]. SIRT3 can increase PPARγ coactivator-1α (PGC-1α) gene expression [73]. In the liver, increased fatty acid oxidation and gluconeogenesis result from SIRT1 activation by resveratrol, NAD^+^, fasting, and calorie restriction, through direct deacetylation of PGC-1α, forkhead box protein O1 (FOXO1), and target of rapamycin complex 2 (TORC2) [74].

In white adipocytes, SIRT1 promotes lipolysis by inhibiting PPARγ through interactions with its cofactors, silencing mediator of retinoid and thyroid hormone receptors (SMRT) and nuclear receptor co-repressor (NCoR) [75]. SIRT1 can also directly deacetylate SREBP, and changes in SIRT1 activity can influence target gene expression, protein stability, and SREBP ubiquitination [76]. OA has been shown to modulate fatty acid oxidation rates by activating the SIRT1-PGC1α transcriptional complex through stimulation of the cAMP-protein kinase A pathway [77]. Additionally, in a rat model of chronic kidney disease, *n*-3 fatty acids have been shown to activate PGC-1α via deacetylation by upregulating SIRT1 and SIRT3 [78].

### 4.2. Inflammatory and Epigenetic Networks

#### 4.2.1. NF-κB Inflammatory Cascade (Central Node for Fatty Acid-Induced Inflammation)

The NF-κB pathway is a central mediator of fatty acid-induced cardiac inflammation. In adipocytes and macrophages, metabolic stress sensors—particularly TLRs—activate the IκB kinase (IKK) complex, which then drives NF-κB-dependent transcription of pro-inflammatory cytokines, including TNFα, IL-1β, and IL-6 [79].

Cardiac consequences of NF-κB activation include fibroblast activation and collagen deposition (driving adverse remodeling after myocardial infarction) and indirect effects via adipocyte lipolysis that release additional FFAs into the circulation [80,81,82].

Specific fatty acid triggers of NF-κB: Palmitic acid activates NF-κB through TLR4 signaling (see Section 5.1 for mechanistic details). By contrast, *n*-3 PUFAs suppress NF-κB via GPR120 and β-arrestin signaling (see Section 4.3.1).

Cross-talk with epigenetic regulation: Histone deacetylase (HDAC) inhibition attenuates pathological gene programs in cardiac fibroblasts and myocytes. For example, HDAC inhibitors reduce hypertrophy and fibrosis [83]. (The role of gut-derived short-chain fatty acids as endogenous HDAC inhibitors is discussed in Section 4.5.4).

#### 4.2.2. MicroRNA Modulation

Furthermore, specific fatty acids can modulate miRNA expression. DHA treatment can alter the expression of miRNAs involved in lipid metabolism and angiogenesis in cardiomyocytes (e.g., miR-107 and miR-223), whereas saturated fat consumption deregulates miRNAs implicated in cardiac hypertrophy and fibrosis [50]. These miRNA shifts directly influence sarcoplasmic/endoplasmic reticulum Ca^2+^-ATPase 2a (SERCA2a) expression and calcium handling in the failing myocardium.

### 4.3. Membrane Receptors and Lipid Signaling

The heart relies on fatty acid-derived signaling for contraction, survival, and adaptation. Key mechanisms include the following:

#### 4.3.1. G-Protein-Coupled Receptor (GPCR) Activation by FFAs

GPCRs (sometimes referred to as seven-transmembrane receptors) are essential membrane proteins expressed on almost every cell type in the body [84]. FFAs act as ligands that bind to and activate FFARs, which are themselves GPCRs [51].

Short-chain FFAs (C2–C6) preferentially activate FFAR2 (GPR43) and FFAR3 (GPR41), whereas FFAR1 (GPR40) senses medium- and long-chain FFAs (>C6) [52,53]. GPR120 is a GPCR that senses medium- and long-chain FFAs and has also been identified alongside FFAR1 [52,85]. FFAR2 can promote cardiomyocyte hypertrophy by activating extracellular signal-regulated kinase 1/2 (ERK1/2), which in turn activates signal transducer and activator of transcription 3 (STAT3). Activation of GATA4 and GPR120 has been associated with improved insulin homeostasis [84,86]. *n*-3 PUFAs preferentially activate GPR120 to inhibit NFκB, whereas PGE_2_ and EP receptors modulate contractility, and leukotriene receptors drive fibrosis [31].

#### 4.3.2. Lipid Raft Modulation

The plasma membrane’s lipid rafts are microdomains rich in sphingolipids and cholesterol that orchestrate and control a range of signaling pathways. Ion channel regulatory proteins and signaling molecules are abundant in lipid rafts, which are also found in cardiac myocytes [87]. Multiple channel types (such as voltage-gated Na^+^, K^+^, and Ca^2+^ channels) and even various isoforms of a single channel are expressed by the majority of cardiovascular system cells, and each channel contributes differently to excitability [88,89].

The effect of natural FFAs on membrane lipid structure depends on their length and degree of unsaturation [90]. For phospholipid bilayers, long-chain SFAs raise the gel-to-fluid phase (Lβ-to-Lα) transition temperature (also called the melting temperature, or Tm). At the same time, short-chain or cis-unsaturated fatty acids (UFAs) lower the Tm [90]. Lipids that contain UFAs increase membrane fluidity because double bonds cause the fatty acid chains to kink, making it harder for them to pack together [91]. Thus, these fatty acids alter the lipid structure of membranes by altering their fluidity, phase behavior, permeability, fusion, lateral pressure, and flip-flop dynamics [92]. DHA partitions into cardiac lipid rafts to modulate Na^+^/Ca^2+^ channel function and reduce the risk of ectopy, while EPA localizes to non-raft regions to optimize anti-arrhythmic effects [49].

#### 4.3.3. Calcium Handling

The connection between the electrical signals that flow through the heart and the contraction of myocytes to pump blood is made possible by calcium (Ca^2+^), a crucial regulator of cardiomyocyte function [93]. During contraction and relaxation, Ca^2+^ is released and reabsorbed through the sarcoplasmic reticulum (SR), an organelle that stores Ca^2+^ [94]. A subtype of the SR/ER Ca^2+^ ATPase expressed in the heart, SERCA2a, mediates cardiomyocyte contraction and the re-entry of Ca^2+^ into the SR from the cytoplasm.

According to an in vitro study conducted at 35 °C, the PUFA content of SR membranes was found to influence cardiac function by modifying SERCA activity in the hearts of hibernating and non-hibernating Syrian hamsters [95]. SERCA activity was adversely affected by DHA levels, but it increased significantly as the percentage of LA in SR phospholipids increased. Another in vitro test revealed that incubation with phosphatidylcholine containing SFAs increased membrane order (stiffness), thereby compromising SERCA activity [96,97]. When the rate of Ca^2+^ reuptake via SERCA2a decreases, Ca^2+^ overload occurs, causing the ventricles to relax slowly or not at all and resulting in diastolic dysfunction [97,98]. 

### 4.4. Mitochondrial Function and Dynamics

#### 4.4.1. Fatty Acid Oxidation Entry

The outer mitochondrial membrane contains the canonical isoforms CPT1A and CPT1B, which carry LCFAs into the mitochondria for β-oxidation [99]. In brief, the heart can absorb fatty acids by diffusion or via FAT and FATP transporters [100]. Fatty acyl-CoA synthase (FACS) within cardiomyocytes esterifies fatty acids bound to FABPs, forming fatty acyl-CoA. CPT1 catalyzes the formation of acylcarnitine, the rate-limiting step in mitochondrial β-oxidation that regulates fatty acid entry into the mitochondria. After transport into the mitochondria, CPT2 converts acylcarnitine back into fatty acyl-CoA, most of which enters the fatty acid β-oxidation cycle [100]. *n*-3-derived EETs from CYP2J2 vasodilate the coronary arteries, thereby matching fatty acid oxidation substrate delivery to cardiac workload [31].

#### 4.4.2. Reactive Oxygen Species (ROS)

During aerobic respiration, mitochondria are the primary consumers of molecular oxygen in cells [101]. Although most oxygen is reduced to water, generating a proton-motive force that drives ATP synthesis [91], a small fraction is partially reduced by the mitochondrial electron transport chain (ETC), producing mitochondrial reactive oxygen species (mtROS) [101]. These mtROS can damage mitochondrial DNA, proteins, and lipids by inducing oxidative stress [102]. Conversely, *n*-3 PUFAs were also found to enhance antioxidant defense against ROS [103]. Treatment with *n*-3 PUFAs prevents dilated cardiomyopathy (DCM) in mice primarily through ROS suppression and mitochondrial protection, though direct pro-apoptotic effects remain uncertain in human disease [104].

#### 4.4.3. Mitochondrial Dynamics: The Fission–Fusion Machinery

Mitochondrial morphology is dynamically regulated by large GTPases of the dynamin superfamily [105]. The key fission promoter is dynamin-related protein 1 (DRP1), which translocates to the outer mitochondrial membrane and mediates organelle division. The key fusion mediators are mitofusin 1 (MFN1), mitofusin 2 (MFN2), and optic atrophy 1 (OPA1) [105,106,107,108,109].

Regulation of DRP1 activity occurs primarily through phosphorylation. Phosphorylation at Ser616 promotes mitochondrial fission, whereas phosphorylation at Ser637 inhibits it. (The pathological consequences of excessive DRP1 activation—particularly in the context of palmitate-induced lipotoxicity—are discussed in Section 5.1; protective effects of DHA via DRP1 inhibition are covered in Section 4.5.2 below).

### 4.5. Emerging Research Frontiers in Cardiac Lipid Biology

#### 4.5.1. Membrane Lipid Remodeling and Microdomain Signaling

##### *n*-3 PUFA Incorporation

EPA and DHA incorporate into membrane phospholipid bilayers, modulate membrane fluidity, and disrupt lipid raft-dependent signaling (e.g., TLR4/NADPH oxidase) [110]. DHA preferentially partitions into lipid rafts, whereas EPA localizes to non-raft regions, which explains their differential anti-inflammatory effects [111]. This differential partitioning influences the localization of COX/LOX receptors and SPM production in cardiac membranes.

##### Cardiolipin Dynamics

Cardiolipin (CL) is a mitochondria-specific phospholipid (approximately 15–20% of inner membrane lipids) that, in the heart, is unusually homogeneous—largely tetralinoleoyl (all 18:2) in composition [112]. It anchors the electron transport supercomplexes and maintains cristae architecture; perturbations in CL quantity or acyl-chain composition dramatically impair mitochondrial bioenergetics and dynamics [112]. Beyond energy metabolism, injured cardiomyocytes externalize oxidized CL, a potent damage-associated molecular pattern that activates innate immune receptors and triggers inflammation [113]. In sum, both the fatty acyl chains of energy substrates and the polar headgroup lipids of membranes integrate to regulate mitochondrial function, calcium handling, and inflammatory signaling in the myocardium.

##### Very Long-Chain SFA Paradox

VLSFAs (C > 24 saturated fatty acids) and their ceramide and sphingomyelin derivatives are inversely correlated with heart failure risk, likely via sphingolipid-mediated membrane stabilization of oxylipin receptors [114], suggesting that these lipids promote healthy myocardial aging. 

#### 4.5.2. Mitochondrial Plasticity and Metabolic Flexibility

##### Fission–Fusion Balance (Protective Effects of *n*-3 PUFAs)

*n*-3 PUFAs markedly influence mitochondrial dynamics and calcium homeostasis. DHA has been shown to stabilize mitochondrial networks under stress by inhibiting Drp1 activation (reducing phospho-Drp1 at Ser616), thereby preventing stress-induced fragmentation, preserving ATP production, and reducing mtROS in cardiac ischemia models [115].

##### Lipotoxic Fission (Cross-Referenced, Not Re-Described)

As detailed in Section 5.1, prolonged palmitate exposure (>8 h) increases ROS production and promotes mitochondrial fission through DRP1 Ser616 phosphorylation, ultimately leading to apoptosis [115,116]. The accumulation of lipids in cardiomyocytes is thus directly coupled to oxidative stress and mitochondrial fragmentation [117].

##### Alternative Fuels

Microbiota-derived short-chain fatty acids (SCFAs such as acetate, propionate, and butyrate) enter the circulation and serve as alternative fuels. In fact, they may contribute to myocardial energetics under specific pathological conditions, although quantitative estimates and their relevance to lipid mediator signaling remain uncertain [83]. Thus, dietary and microbial lipids together tune mitochondrial metabolism and calcium signaling in cardiomyocytes.

#### 4.5.3. Chrononutrition and Circadian Lipid Metabolism

##### Meal Timing

Chrononutrition (which examines the relationship between the timing of food intake and the body’s circadian rhythms) has emerged as a key variable: recent cohort analyses indicate that the circadian timing of fat intake influences cardiovascular risk. For instance, Evening PUFA intake lowers CVD mortality (HR 0.85), while breakfast PUFA increases risk (HR 1.30) [118]. These associations suggest circadian regulation of COX-2/SPM biosynthesis, though RCTs are needed to establish causality. One speculative explanation is that evening *n*-3 PUFA intake may better align with circadian regulation of SPM biosynthesis, whereas morning intake could interfere with diurnal patterns of arachidonic acid mobilization; however, direct evidence linking meal timing to cardiac oxylipin flux is currently lacking. 

##### Time-Restricted Feeding

Time-restricted feeding aligns nutrient intake with circadian lipid oxidation cycles, improving plasma triglycerides, HDL, and myocardial FAO gene expression in metabolic syndrome models [119].

#### 4.5.4. Gut–Heart Axis Modulation

##### SCFAs as Epigenetic Modulators

Although SCFAs are not lipid mediators in the classical prostaglandin or leukotriene sense, they may indirectly influence cardiac inflammatory tone by shaping the epigenetic and metabolic contexts in which oxylipin signaling operates. Microbial SCFAs have been shown to exert epigenetic effects: butyrate and propionate inhibit host HDACs [83]. This HDAC inhibition has functional consequences: it attenuates pathological gene programs in cardiac fibroblasts and myocytes. Indeed, studies show that HDAC inhibitors (including SCFAs such as butyrate and valproate) reduce hypertrophy and fibrosis by altering chromatin at growth factor target genes. SCFAs have been reported to dampen oxidative and proinflammatory signaling, thereby indirectly influencing the inflammatory milieu in which oxylipin signaling operates [83]. Collectively, SCFAs may modulate the cellular context in which *n*-3–derived specialized pro-resolving mediators exert cardioprotective effects, without directly participating in oxylipin biosynthesis.

##### TMAO Counteraction

The gut–heart axis is also a significant research frontier: shifts in microbiota composition alter circulating lipids (low SCFAs, high trimethylamine N-oxide (TMAO)), thereby modulating vascular tone and inflammation. Mouse trials have shown that restoring SCFAs (via fiber or acetate) lowers blood pressure and attenuates cardiac remodeling [83,120]. Direct evidence linking gut-derived signals to myocardial oxylipin production or to prostaglandin- and leukotriene-mediated cardiac outcomes remains limited.

#### 4.5.5. Omics-Driven Discoveries

##### Lipidomics

Integrative lipidomic and transcriptomic approaches are mapping the cardiac lipidome and its genetic regulation. For example, a recent “lipidome atlas” of the developing heart revealed that postnatal enrichment of DHA-containing phosphatidylcholines and phosphatidylethanolamines coincides with upregulation of specific acyltransferases (Lpcat3 and Agpat3) [121], pinpointing molecular drivers of membrane remodeling. In adult studies, combined omics profiling is uncovering lipid signatures and gene modules that distinguish healthy from diseased myocardium. In sum, emerging chronobiology and multi-omics tools are deepening our mechanistic understanding of how diverse fatty acids regulate cardiac function and adaptation [118,121].

##### Transcriptomics

PPARα-KLF5/15 networks coordinate FAO, and KLF4 deletion impairs mitochondrial biogenesis [36,37,38]. In sum, emerging chronobiology and multi-omics tools are deepening our mechanistic understanding of how diverse fatty acids regulate cardiac function and adaptation [118,121].

In summary, the molecular pathways linking fatty acids to cardiac function span transcriptional regulation (PPARs, SIRTs, AMPK), inflammatory signaling (NF-κB, miRNAs), membrane receptor activation (GPCRs, lipid rafts), and mitochondrial dynamics (fission–fusion, ROS). Emerging frontiers—including chrononutrition, the gut–heart axis, and multi-omics approaches—are revealing new layers of complexity. A unifying theme is that the balance between *n*-6 and *n*-3 PUFA derivatives (pro-inflammatory vs. pro-resolving mediators) and between SFA-induced lipotoxicity and *n*-3-mediated protection determines whether fatty acids support or impair cardiac health.

## 5. Pathological Effects of Fatty Acids

Fatty acids have differential effects on cardiac health depending on their structure and metabolic context. While UFAs, particularly *n*-3 PUFAs, are often cardioprotective, excessive intake of SFAs and TFAs is closely linked to adverse cardiac outcomes. This section outlines key pathological mechanisms by which fatty acids contribute to cardiovascular disease, with an emphasis on how the beneficial and harmful effects described in Section 3 and Section 4 become dysregulated under conditions of overload or imbalance.

### 5.1. Lipotoxicity and Metabolic Stress: Central Role of Ceramide Signaling

When fatty acid influx exceeds the heart’s β-oxidation capacity, toxic lipid intermediates accumulate—most notably ceramides and diacylglycerols (DAGs). These intermediates impair insulin signaling, activate PKCθ, and trigger apoptosis through ER stress and UPR pathways [55]. The molecular pathway linking excess palmitate to cardiomyocyte death has been well characterized. Palmitate undergoes de novo ceramide synthesis via serine palmitoyltransferase. This leads to activation of protein phosphatase 2A (PP2A), subsequent dephosphorylation and inactivation of Akt, nuclear translocation of FOXO3a, and transcriptional induction of pro-apoptotic mediators, including Bim and Bad [10]. (For the downstream effects of this pathway on mitochondrial fission via DRP1, see Section 4.5.2).

The clinical consequences of ceramide accumulation include diastolic dysfunction, systolic decline, and fibrosis—a phenotype termed lipotoxic cardiomyopathy. These conditions are exacerbated by diabetes and obesity, where CD36 and FAT upregulation amplify LCFA uptake [33].

In addition to ceramide-driven damage, palmitic acid activates TLR4 and NFκB signaling, which promotes atherogenesis and further cardiac inflammation. For a detailed description of the NFκB pathway, its activation by TLRs, and its downstream cardiac consequences (including fibroblast activation and collagen deposition), see Section 4.2.1 [79,110,122,123].

Several counter-regulatory mechanisms mitigate ceramide toxicity. *n*-3 PUFAs (EPA and DHA) attenuate ceramide accumulation by activating PPARα-dependent peroxisomal oxidation pathways, including COX2 upregulation [40,41,42,43,44]. EPA-derived PGE_3_ competitively antagonizes AA-derived PGE_2_ signaling at EP2 and EP4 receptors, thereby suppressing TGFβ-driven cardiac fibroblast activation [110]. By contrast, the *n*-6-derived LTB_4_ (produced via 5-LOX) exacerbates macrophage infiltration and collagen deposition following injury [79,122,123].

In synthesis, the balance between ceramide generation (driven by excess SFAs, particularly palmitate) and ceramide clearance (enhanced by *n*-3 PUFAs via PPARα) is a critical determinant of whether fatty acid overload results in adaptive remodeling or lipotoxic heart failure. Clinically, this suggests that dietary interventions that reduce palmitate intake (e.g., limiting red meat and palm oil) while increasing *n*-3 PUFA intake (e.g., from oily fish) may synergistically protect against lipotoxic cardiomyopathy.

### 5.2. Fatty Acid Profiles in Heart Failure Phenotypes

Systolic failure (heart failure with reduced ejection fraction, HFrEF) is marked by reduced fatty acid oxidation, increased glycolysis via pyruvate dehydrogenase kinase 4 (PDK4), impaired CPT1 activity, and ceramide accumulation [55]. PPARα and PGC-1α repression reduce mitochondrial biogenesis; partial fatty acid oxidation inhibition (with drugs such as trimetazidine or ranolazine) restores coupling efficiency [124].

Paradoxically, in diastolic impairment (heart failure with preserved ejection fraction, HFpEF), fatty acid oxidation can increase despite fibrosis and stiffness, with palmitate activating NLRP3 and IL-1β, contributing to diastolic dysfunction [41]. *n*-3 trials show mixed effects on ejection fraction preservation but a consistent reduction in sudden death [125].

Distinct oxylipin profiles characterize these phenotypes. HFrEF shows an *n*-6/*n*-3 ratio exceeding 15:1, with elevated AA-derived hydroxyeicosatetraenoic acids (HETEs) and LTB_4_. HFpEF shows low EPA and DHA levels that correlate with fibrosis severity [126]. Resolvin D1 infusion improves diastolic parameters in preclinical HFpEF models [127].

In synthesis, the failing heart is not simply deficient in fatty acid oxidation; rather, the balance between *n*-6 and *n*-3 derivatives shifts toward pro-inflammatory and pro-fibrotic mediators. This suggests that therapeutic strategies must consider both the quantity and the quality (*n*-6/*n*-3 ratio) of fatty acids available to the failing myocardium.

### 5.3. Ischemia–Reperfusion Injury and Arrhythmia

During acute ischemia, AA release increases, causing a COX-2-mediated imbalance between thromboxane A_2_ (TXA_2_) and prostacyclin that promotes coronary vasoconstriction and thrombosis [128,129,130]. Reperfusion elevates LTB_4_, activating neutrophils and contributing to microvascular no-reflow [131,132].

*n*-3 PUFAs exert protective effects in this context. Acute infusion of *n*-3 PUFAs produces protectin D1, reducing infarct size by 30–50% via ALX/FPR2-mediated neutrophil apoptosis [133,134]. DHA incorporation into the sarcolemma shortens action potential duration by directly modulating the sodium channel Nav1.5 and SERCA2a [33,51,52,53], whereas high-dose EPA or DHA can increase the risk of atrial fibrillation by hyperpolarizing the ultra-rapid delayed rectifier potassium current (IKur) [4]. TFAs disrupt connexin-43 localization, leading to conduction heterogeneity [28,29].

In synthesis, the outcome of ischemia–reperfusion is determined by the balance between AA-derived pro-thrombotic and pro-inflammatory mediators (TXA_2_, LTB_4_) and *n*-3-derived pro-resolving mediators (protectin D1, resolvins). This balance can be modulated by dietary fatty acid intake and by acute *n*-3 administration.

### 5.4. Atherosclerosis and Vascular Interactions

Cardiac capillary endothelial cells express COX-2 and 5-LOX, and the resulting PGE_2_ and LTB_4_ promote monocyte adhesion and facilitate LPL-mediated delivery of LCFAs to foam cells [135,136]. *n*-3 SPMs (e.g., maresin-1) activate macrophage efferocytosis (the clearance of apoptotic cells), thereby improving plaque stability [110]. Perivascular adipose tissue releases palmitate, which activates cardiac TLR4 and drives concentric left ventricular remodeling [137].

In synthesis, fatty acids influence not only cardiomyocyte function but also the vascular and inflammatory environment of the heart. The shift from AA-derived to *n*-3-derived mediators reduces monocyte adhesion, enhances plaque stability, and limits perivascular inflammation.

These findings underscore the importance of dietary modulation (Table 1)—specifically, reducing SFA and TFA intake and increasing *n*-3 PUFA intake—as a strategy to prevent and mitigate cardiac disease.

## 6. Clinical Implications and Epidemiological Studies

Among the four major fatty acid classes, trans fats and most SFAs increase coronary heart disease (CHD) risk by raising serum cholesterol levels [141,142]. However, the relationship between SFAs and cardiovascular disease (CVD) is not monolithic; it depends critically on chain length and food source. A 14-year follow-up study (*n* = 939 incidents) found that longer-chain SFAs (12:0–18:0) were independently associated with higher CHD risk, whereas short- to medium-chain SFAs (4:0–10:0) were not [143]. Strikingly, very-long-chain SFAs (VLSFAs; >C24), such as lignoceric acid, exhibit cardioprotective effects—higher circulating levels are associated with lower risks of heart failure, CHD, atrial fibrillation, and all-cause mortality [114]. These opposing effects by chain length are mechanistically consistent: longer-chain SFAs (C12–C18) displace AA from membrane phospholipids, increasing production of pro-thrombotic TXA_2_ and pro-inflammatory PGE_2_, whereas VLSFAs (>C24) stabilize oxylipin receptor microdomains within lipid rafts, potentially enhancing anti-inflammatory signaling [114,144,145].

The EPIC-CVD case–cohort study (*n* = 385,747; 10,529 CHD cases) showed that SFA effects depend critically on food sources rather than on the fatty acids in isolation [146]. Harmful sources included red meat (HR: 1.07; 95% CI: 1.02–1.12) and butter (HR: 1.02; 95% CI: 1.00–1.04) [146]. By contrast, neutral or beneficial sources included yogurt (HR: 0.93; 95% CI: 0.88–0.99), cheese (HR: 0.98; 95% CI: 0.96–1.00), and fish (HR: 0.87; 95% CI: 0.75–1.00) [146]. This source-dependent pattern is further supported by Dutch cohort data, which show that higher palmitic acid intake and substitution of SFAs with animal protein increase CHD risk [144]. Plant-derived SFAs may raise LDL cholesterol (3.43–9.18 mg/dL) but also increase HDL cholesterol (0.94–1.89 mg/dL), yielding neutral cardiovascular effects when substituted for plant-derived UFAs [145]. The apparent paradox—that some SFA-rich foods such as cheese and yogurt do not increase CVD risk despite raising LDL cholesterol—is resolved by considering the accompanying nutrients. Fermented dairy contains vitamin K_2_, probiotics, and bioactive peptides that may offset LDL cholesterol elevation through anti-inflammatory and endothelial-protective effects. Moreover, the physical matrix of cheese (calcium-bound fatty acids) reduces postprandial lipemia compared with butter. Notably, a comprehensive review of studies from 2010 to 2021 found no consistent correlation between naturally occurring SFA consumption and CVD risk [147]. This does not mean SFAs are harmless; rather, it means that the effect of SFAs is entirely context-dependent—on chain length, food matrix, and what nutrient replaces them (replacement with refined carbohydrates is harmful; replacement with PUFAs is beneficial).

UFAs also exhibit complex and sometimes contradictory cardiometabolic effects. A 12-year study of 1807 ischemic heart disease (IHD) cases paradoxically found that replacing SFAs with cis-MUFAs, PUFAs, or animal protein increased the risk of IHD (HR per 5% energy: 1.27–1.37) [148]. At first glance, this contradicts the widely accepted benefit of replacing SFAs with UFAs. However, this apparent contradiction is resolved by examining the type of UFA and the population context. The harmful substitution in the Dutch study involved replacing dairy SFAs (which, as noted above, may be neutral or beneficial due to food matrix effects) with animal-derived MUFAs and PUFAs from processed meats—not with plant-based UFAs from olive oil, nuts, or fish. By contrast, meta-analyses indicate that MUFAs reduce total and hemorrhagic stroke risk [149], although they do not consistently reduce CVD mortality [150]. Furthermore, replacing SFAs with UFAs, carbohydrates, or protein reduces CVD events by 21% [151], and MUFAs and PUFAs similarly improve triglycerides, LDL cholesterol, and blood pressure—though MUFAs show superior nighttime systolic blood pressure reduction, a critical CVD risk factor [152,153].

The cardioprotective effects of UFAs are mechanistically explained by optimization of the tissue *n*-6/*n*-3 balance. Marine *n*-3 PUFAs (EPA and DHA) generate anti-arrhythmic series-3 prostaglandins (PGE_3_) and SPMs (resolvins and protectins) that compete with AA-derived pro-thrombotic TXB_2_ and pro-inflammatory LTB_4_. This competition explains why *n*-3 PUFAs reduce sudden cardiac death even when their effects on overall CVD mortality are modest or inconsistent across trials [141,154,155,156,157,158,159]. The inconsistent results across UFA trials likely reflect differences in baseline *n*-6/*n*-3 ratios, with greater benefits observed in populations with high baseline *n*-6 intake (Western diets, *n*-6/*n*-3 > 10:1) and minimal benefits in those already consuming balanced ratios (<4:1).

### 6.1. Food Source Considerations

The source of fatty acids substantially modulates their cardiovascular impact. International guidelines emphasize:

Beneficial effects are associated with plant-based foods (whole grains, fruits, vegetables), sea fish, fermented dairy, and lean meats [160].

Detrimental effects are associated with processed meats, sugar-sweetened beverages, butter, cream, and refined starches [160]. Whole-food approaches (e.g., the Mediterranean diet) reduce CVD mortality more effectively than isolated nutrient modifications [161].

Sea fish provide EPA and DHA, which increase SPMs (resolvins and protectins); plant sources optimize LA and ALA, resulting in tissue *n*-6/*n*-3 ratios below 5:1 and reduced production of pro-inflammatory eicosanoids [162,163,164].

### 6.2. Timing of Intake

Emerging evidence highlights meal timing as a key modulator of the effects of UFAs. In a cohort of 30,136 adults, high breakfast intake of PUFAs (HR: 1.30; 95% CI: 1.13–1.50), MUFAs (HR: 1.28; 95% CI: 1.13–1.45), or total UFAs (HR: 1.35; 95% CI: 1.17–1.57) increased CVD mortality. Conversely, dinner consumption of MUFAs/total UFAs reduced all-cause mortality, while PUFAs lowered both CVD and all-cause mortality [118]. However, these observations are derived from association studies and do not establish causality, as residual confounding and reverse causation cannot be excluded. Evening *n*-3 intake aligns with peak SPM biosynthesis; breakfast disrupts diurnal AA mobilization, which increases pro-thrombotic eicosanoids [165,166].

### 6.3. Reconciling Controversies: A Unifying Perspective

The relationship between fatty acids and CVD is not monolithic. SFA effects depend critically on chain length (with VLSFAs protective) and on the food matrix (dairy sources being neutral or beneficial vs. meat sources being harmful). UFAs generally confer benefits, but outcomes vary by type (*n*-3 > *n*-6 PUFAs), source (plant > animal), and timing (dinner > breakfast). These nuances explain apparent contradictions in the literature and underscore the importance of considering fatty acids within their dietary context rather than as isolated nutrients. Clinical heterogeneity reflects tissue *n*-6/*n*-3 ratios, which shape the oxylipin repertoire (pro-thrombotic series-2 vs. anti-arrhythmic series-3 and SPMs) [167]. VLSFAs stabilize receptor microdomains; meal timing modulates circadian PLA2/COX-2 activity.

In clinical summary, for cardiovascular prevention, the evidence supports the following hierarchy of dietary fat substitutions:

- Most beneficial: Replace SFAs from red meat and butter with PUFAs from fish (EPA and DHA), nuts, and plant oils, or with MUFAs from olive oil and avocados.
- Neutral (context-dependent): Dairy-derived SFAs from cheese and yogurt do not increase CVD risk, likely due to food matrix effects and fermentation products.
- Harmful: Replace SFAs with refined carbohydrates or with animal-derived UFAs from processed meats.
- Chain length matters: VLSFAs (>C24) from fish and dairy are cardioprotective; longer-chain SFAs (C12–C18) from palm oil and red meat are harmful.
- Timing matters (emerging): Evening consumption of UFAs appears more beneficial than morning intake, possibly due to circadian regulation of oxylipin biosynthesis, though randomized trials are needed to confirm causality.

## 7. Interventional Studies

Evidence-based dietary patterns consistently show cardioprotective effects, though their benefits are multifaceted and cannot be attributed solely to fatty acid composition [168,169,170]. This section reviews the major dietary patterns and supplementation strategies tested in clinical trials, with an emphasis on the mechanistic basis for their effects (drawing on the pathways described in Section 3 and Section 4) and the clinical outcomes observed.

### 7.1. The Mediterranean Diet

The Mediterranean diet is characterized by high intake of extra-virgin olive oil (EVOO), nuts, legumes, whole grains, and vegetables. In the PREDIMED trial, supplementing with EVOO or nuts reduced major cardiovascular events compared with a low-fat control diet [170]. EVOO provides OA and polyphenols that favor competition between LA and ALA, resulting in tissue *n*-6/*n*-3 ratios of approximately 4:1, which increases resolvin production and downregulating LTB_4_, helping to explain the 30% reduction in cardiovascular events observed in PREDIMED [167,170,171].

### 7.2. The DASH Diet

The Dietary Approaches to Stop Hypertension (DASH) diet, which emphasizes fruits, vegetables, low-fat dairy, and reduced sodium, robustly lowers blood pressure and improves lipid profiles [168,169]. ALA enrichment, combined with low SFA intake, increases circulating EPA, promoting series-3 prostaglandins and complementing potassium-mediated vasodilation [172,173].

### 7.3. Plant-Based Diets

Similarly, plant-based diets rich in whole foods and fiber are associated with lower LDL cholesterol levels, reduced inflammation, and a lower incidence of CHD [174,175,176]. LA and ALA synergy drives *n*-6/*n*-3 ratios below 5:1, reducing TXB_2_ levels and thrombosis propensity despite limited DHA conversion from ALA [177,178].

In synthesis, the mechanisms underlying the success of these diets are complex and synergistic. While the high content of MUFAs and PUFAs in the Mediterranean diet likely contributes to improved lipid profiles and reduced inflammation, other components—such as fiber, polyphenols, antioxidants, and low glycemic load—also play significant, potentially additive roles in reducing oxidative stress, improving endothelial function, and modulating the gut microbiome [179]. Therefore, the cardioprotection offered by these patterns is best viewed as an emergent property of the entire dietary matrix, rather than a consequence of any single nutrient class—though tissue *n*-6/*n*-3 ratios and oxylipin profiles provide mechanistic biomarkers of their efficacy [180].

### 7.4. n-3 PUFA Supplementation Trials

Several clinical trials have examined the effects of *n*-3 PUFA supplementation on CVD outcomes. Table 2 summarizes the key trials.

In synthesis, these trials demonstrate that *n*-3 PUFAs reduce cardiovascular mortality and sudden death, particularly in secondary prevention populations. However, as noted in Section 1, high-dose pharmaceutical formulations (≥4 g/day) have been associated with increased atrial fibrillation risk in some trials (REDUCE-IT, STRENGTH, VITAL-Rhythm). This dose-dependent dual effect—reduced mortality but increased arrhythmia risk—highlights the need for careful patient selection and dosing strategies.

### 7.5. Integrating Dietary Patterns with Pharmacotherapy

Growing evidence indicates that combining *n*-3 PUFA supplementation with statin therapy yields incremental benefit. A recent meta-analysis of imaging studies found that patients receiving both high-dose EPA and DHA and a statin (compared with statin alone) had significantly slower progression of coronary plaque, thicker fibrous caps, and lower high-sensitivity C-reactive protein (hs-CRP) levels [181]. In other words, *n*-3 PUFAs appeared to stabilize atherosclerotic lesions beyond the effects of statins alone, without adverse changes in HDL or LDL cholesterol levels. Mechanistically, both statins and *n*-3 PUFAs share anti-inflammatory and plaque-modulating pathways (for example, each promotes the production of pro-resolving lipid mediators) [182]. These synergistic effects translate into improved lipid profiles (notably lower triglycerides) and attenuated vascular inflammation when diet and drug are combined.

In synthesis, a comprehensive cardioprotective diet emphasizes vegetables, fruits, whole grains, legumes, nuts, fish, and unsaturated fats while moderating refined carbohydrates and saturated fats, with special attention to fat sources (dairy vs. meat) and fat types (MUFA vs. PUFA) [170,183,184]. Emerging strategies, such as time-restricted feeding and increased intake of oily fish, further improve traditional risk factors (blood pressure, dyslipidemia, endothelial function, and inflammatory biomarkers) [182,185]. When combined with optimal pharmacotherapy, these dietary approaches can yield additive cardiometabolic benefits. For instance, co-administering *n*-3 PUFAs with statins yields greater reductions in triglycerides, hs-CRP, and plaque vulnerability than statins alone [181,182]. Collectively, these data support a multifaceted dietary prescription (e.g., Mediterranean, DASH, or plant-based patterns with source-specific fat guidance and consideration of meal timing) to maximize cardiovascular prevention and treatment outcomes.

## 8. Future Directions

As detailed throughout this review, recent advances in membrane lipid remodeling (e.g., VLSFAs) and gut–heart axis signaling provide novel targets for intervention. This section outlines future therapeutic directions, innovative dietary strategies, and emerging research tools that may transform the management of fatty acid-related cardiac diseases.

### 8.1. Future Therapeutic Directions

Modulating fatty acid uptake is a logical strategy to prevent lipotoxicity in the heart. In cardiomyocytes, LCFAs enter the cell via LPL cleavage products and surface transporters, including FAT/CD36, FABPpm, and members of the FATP family [186]. Notably, transgenic mice with cardiac-specific overexpression of FATP1 exhibit massive lipid uptake and develop lipotoxic cardiomyopathy [186]. At the same time, FAT/CD36 deficiency shifts myocardial substrate use toward glucose and can protect against lipid overload. Conversely, angiopoietin-like protein 4 (ANGPTL4)—an LPL inhibitor induced by PPARδ—limits lipid uptake into cells [176] and has been shown to mitigate lipid accumulation in muscle and heart.

However, therapeutic inhibition of FAT/CD36 may not be without risk: FAT/CD36 facilitates rapid fatty acid uptake during cardiac stress, and its absence may impair myocardial energy homeostasis under increased workload [187,188,189,190]. Thus, careful targeting—such as partial or context-dependent modulation rather than complete inhibition—may be necessary to avoid compromising cardiac performance.

FFAR4 and GPR120 agonists (*n*-3 PUFAs) inhibit NFκB through β-arrestin and sequester NLRP3 in cardiomyocytes [191,192,193,194]. Developing more selective and potent FFAR4 agonists that do not carry the off-target effects of high-dose *n*-3 PUFAs (such as increased AFib risk) represents a promising pharmacological avenue.

Once inside the cell, fatty acids must be processed by mitochondrial and enzymatic machinery to prevent toxic buildup. CPT1 controls mitochondrial entry of LCFAs; its inhibition by malonyl-CoA (produced by ACC) provides a checkpoint for fatty acid oxidation. Thus, ACC inhibitors or malonyl-CoA decarboxylase (MCD) activators can enhance CPT1 activity and boost β-oxidation, improving energy output. At the gene level, PPAR nuclear receptors orchestrate these pathways: PPARα (with coactivator PGC-1α) upregulates genes for fatty acid transport and β-oxidation (including CPT1), whereas PPARδ similarly promotes oxidative metabolism. Activators of PPARα and PPARδ, as well as SIRT1 and SIRT3, increase CPT1 and MCAD expression while reducing SREBP-1c, thereby accelerating fatty acid oxidation [76,195]. These combined interventions—spanning fatty acid transporters, metabolic enzymes, nuclear receptors, and immune sensors—form a coherent therapeutic framework to prevent lipotoxic cardiac injury and improve substrate handling in CVD.

In synthesis, future pharmacological strategies should move beyond simply increasing or decreasing fatty acid flux. Instead, precision targeting of specific transporters (partial CD36 inhibition), receptors (FFAR4-selective agonists), and metabolic enzymes (ACC inhibitors, MCD activators) may achieve the dual goal of reducing lipotoxicity while preserving the beneficial signaling and energy functions of fatty acids.

### 8.2. Innovative Dietary Strategies

While established dietary patterns (Mediterranean, DASH, and plant-based diets) have proven cardioprotective effects (see Section 7), emerging concepts in diet timing and composition warrant further investigation.

Chrononutrition suggests that restricting the daily eating window may improve cardiometabolic health. In a trial of patients with metabolic syndrome, adopting a 10 h self-selected feeding window (TRE) for 12 weeks led to weight loss, lower blood pressure, and reductions in atherogenic lipid fractions [185]. Thus, TRE can complement standard medical care by improving risk factors without calorie counting. However, the effects of TRE on cardiac oxylipin profiles and SPM biosynthesis have not been directly studied.

Meal timing of fat intake also requires further investigation. As noted in Section 6.2, cohort analyses suggest that evening PUFA intake lowers CVD mortality, whereas breakfast PUFA intake increases risk [118]. These associations require confirmation in randomized controlled trials with mechanistic endpoints (e.g., circadian patterns of COX-2 and SPM biosynthesis).

Gut microbiome modulation represents another frontier. While SCFAs (produced by microbial fermentation of fiber) have been shown to exert HDAC-inhibitory and anti-inflammatory effects [83], direct evidence linking gut-derived signals to myocardial oxylipin production remains limited. Future studies should test whether fiber or SCFA supplementation enhances the cardioprotective effects of *n*-3 PUFAs.

Finally, integrating dietary strategies with pharmacotherapy (discussed in Section 7.5) warrants further investigation. The synergistic effects of *n*-3 PUFAs and statins on plaque stabilization [181] suggest that other combinations (e.g., TRE plus FFAR4 agonists or SCFA supplementation plus EPA/DHA) may yield additive benefits.

In summary, future research should prioritize randomized controlled trials of chrononutrition strategies (TRE, evening PUFA intake), gut microbiome modulation (fiber, SCFAs), and dietary–pharmacological combinations, with mechanistic endpoints including oxylipin and SPM profiles.

## 9. Conclusions

Longer-chain SFAs (C14:0–C18:0), especially those found in red meat, butter, and palm oil, are consistently associated with increased CVD risk, particularly CHD. These associations reflect the fact that palmitate displacement of AA from membrane phospholipids is linked to increased pro-thrombotic TXA_2_ and PGE_2_ production [110]. Although associations between other SFAs and UFAs and specific CVD outcomes remain inconsistent, this likely reflects residual confounding and heterogeneity across food sources, metabolism, and clinical context.

Importantly, not all SFA-rich foods are equal. While processed meats, butter, and cream should be minimized—especially for individuals with diabetes or elevated CVD risk—fermented dairy (e.g., yogurt, cheese) and seafood (e.g., sardines) often have neutral or even beneficial cardiometabolic effects due to their complex nutrient profiles. VLSFAs from dairy and fish stabilize oxylipin receptor microdomains, while seafood delivers EPA and DHA, which produce cardioprotective series-3 prostaglandins and SPMs [196,197,198].

Replacing SFAs with PUFAs, particularly those from whole foods such as fish, nuts, and olive oil, remains a cornerstone dietary strategy for CVD prevention. Substituting SFAs with EPA and DHA, which is associated with a lower tissue *n*-6/*n*-3 ratio, has been linked to reduced dominance of AA-derived LTB_4_ and TXB_2_ signaling and may contribute to the observed reduction in cardiovascular events [180]. Evidence-based patterns such as the Mediterranean and DASH diets—rich in these components—should be prioritized across populations, including those with established CVD. By contrast, dietary strategies such as the ketogenic diet (KD) and intermittent fasting (IF) fall outside the core scope of this review; their effects on cardiac EFA pools and oxylipin signaling remain insufficiently explored and warrant future investigation.

Whole foods should be prioritized over supplements. For instance, sardines and other oily fish provide *n*-3 PUFAs along with beneficial nutrients such as calcium, potassium, and selenium, making them superior to isolated supplements. While *n*-3 PUFA supplementation may be beneficial in some cases, it is limited by unclear dosing thresholds, bioavailability issues, and potential risks (e.g., an increased risk of atrial fibrillation at high doses).

In summary, fatty acids play dual roles in cardiac physiology and pathology through distinct metabolic and signaling pathways. Cardioprotective effects depend not only on fatty acid type but also on dietary context, balance, and metabolic state.

### 9.1. Main Findings

The following key conclusions emerge from this review:

First, the cardiovascular impact of fatty acids cannot be predicted from saturation alone. Chain length (VLSFAs protective vs. longer-chain SFAs harmful), double-bond geometry (cis vs. trans), and the *n*-6/*n*-3 ratio collectively determine whether fatty acids promote inflammation and thrombosis or support resolution and cardiac protection.

Second, *n*-3 PUFAs (EPA and DHA) reduce cardiovascular mortality and sudden death through multiple mechanisms, including anti-arrhythmic effects (modulation of sodium and calcium channels), anti-inflammatory effects (GPR120-mediated NFκB suppression), and pro-resolving effects (generation of SPMs such as resolvins and protectins). However, high-dose formulations (≥4 g/day) increase atrial fibrillation risk, necessitating careful patient selection.

Third, SFAs are not a homogeneous class. Palmitic acid (C16:0) from red meat and palm oil promotes ceramide-driven lipotoxicity and TLR4/NFκB-mediated inflammation, whereas stearic acid (C18:0) is rapidly converted to oleic acid and exerts neutral effects. VLSFAs (>C24) from dairy and fish are associated with lower CVD risk, likely through stabilization of oxylipin receptor microdomains.

Fourth, the food matrix fundamentally alters the effects of fatty acids. Fermented dairy (cheese, yogurt) does not increase CVD risk despite containing SFAs, due to accompanying nutrients (vitamin K_2_, probiotics, bioactive peptides) and physical matrix effects (calcium-bound fatty acids reducing postprandial lipemia).

Fifth, meal timing of fat intake may influence cardiovascular outcomes, with evening consumption of UFAs appearing more beneficial than morning intake, possibly due to circadian regulation of COX-2 and SPM biosynthesis. However, these associations require confirmation in randomized controlled trials.

### 9.2. Future Research Directions

Based on the findings of this review, the following research priorities are identified:

First, develop and test selective FFAR4 (GPR120) agonists that mimic the beneficial effects of *n*-3 PUFAs (NFκB suppression, NLRP3 inhibition) without the off-target effects of high-dose EPA and DHA (increased AFib risk).

Second, evaluate partial CD36 inhibitors or context-dependent modulators that reduce lipotoxicity in obesity and diabetes without compromising stress-induced fatty acid uptake, which is necessary for cardiac energy production.

Third, conduct randomized controlled trials of chrononutrition strategies (time-restricted eating, evening PUFA intake) with hard cardiovascular endpoints and mechanistic outcomes (circadian oxylipin and SPM profiles).

Fourth, integrate multi-omics approaches (lipidomics, transcriptomics, metabolomics) to identify patient subgroups most likely to benefit from specific dietary or pharmacological interventions based on baseline *n*-6/*n*-3 ratios, genetic variants (e.g., FADS1/2 polymorphisms), and gut microbiome composition.

Fifth, establish whether gut microbiome modulation (e.g., through fiber or SCFA supplementation) can enhance the cardioprotective effects of *n*-3 PUFAs and SPMs, and whether gut-derived signals directly influence myocardial oxylipin production.

Sixth, compare whole-food sources of *n*-3 PUFAs (e.g., sardines, walnuts, flaxseed) with isolated supplements in head-to-head trials with mechanistic endpoints (tissue *n*-6/*n*-3 ratios, oxylipin profiles, and hard cardiovascular outcomes).

In the final summary, fatty acids are not simply fuels for the heart; they are signaling molecules that shape inflammation, excitability, and remodeling through complex networks of oxylipins and SPMs. Optimizing cardiac fatty acid metabolism requires a holistic approach that considers fatty acid type, chain length, food source, dietary pattern, meal timing, and individual metabolic context. Future research should prioritize precision nutrition strategies that integrate multi-omics profiling with targeted dietary and pharmacological interventions to maximize cardiovascular protection while minimizing risks such as atrial fibrillation and lipotoxicity.

## Figures and Tables

**Table 1:** Summary of pathogenic mechanisms and CVD outcomes by fatty acids.

Fatty Acid Class	Key Examples	Oxylipin Effects	Cardiac Outcomes
Saturated (SFAs)	Palmitic acid (C16:0) Stearic acid (C18:0)	↑ AA-derived PGE_2_, TXB_2_, LTB_4_ (via membrane displacement of EFAs) ↓ EFA incorporation	↑ Lipotoxicity, inflammation ↑ CHD, HF risk Stearic: neutral [11,40,41,42,43,44].
Monounsaturated (MUFAs)	Oleic acid (C18:1*n*-9)	↓ AA availability (via ELOVL5/6 competition) ↓ Series-2 PGs	↓ NLRP3 activation ↓ Stroke risk Mediterranean diet [19,20,138,139,140]
*n*-6 PUFAs	LA → AA (20:4*n*-6)	↑ Series-2 PGs (PGE_2_, TXB_2_) ↑ LTB_4_ (5-LOX)	Pro-thrombotic ↑ Inflammation (when *n*-6/*n*-3 >10:1) [13,24,25]
*n*-3 PUFAs	ALA → EPA/DHA EPA (20:5*n*-3), DHA (22:6*n*-3)	↑ Series-3 PGs (PGE_3_) ↑ Resolvins, protectins ↓ Series-2 PGs/LTs	↓ CV mortality ↓ Arrhythmias ↑ AF risk (high-dose) [26,27]
Trans (TFAs)	Elaidic acid (18:1*n*-9t)	Disrupts COX/LOX localization ↑ LDL/HDL ratio	↑ Endothelial dysfunction ↑ CHD risk [28,29]

↑ indicates increased production or levels. ↓ indicates decreased production or levels.

**Table 2:** Key Clinical Trials of *n*-3 PUFA Supplementation and CVD Outcomes.

Intervention	Key Trials	Patient Population	Primary Outcome
dietary fish oil, 850 mg EPA/DHA	GISSI-Prevenzione	Post-MI	↓ Sudden death (45%) ↓ Total mortality [27]
fatty fish dietary advice	DART	Men post-MI	↓ All-cause mortality (29%) [159]
EVOO/nuts (ALA source)	PREDIMED	Primary prevention (no CVD)	↓ CV events (30%) [170]

↓ = reduction in risk or events.

## Data Availability

No new data were created or analyzed in this study.

## Abbreviations

AA: arachidonic acid; ACC: acetyl-CoA carboxylase; ACOX1: acyl-CoA oxidase 1; ADF: alternate day fasting; Agpat3: 1-acylglycerol-3-phosphate O-acyltransferase 3; ALA: α-linolenic acid; AMPK: AMP-activated protein kinase; ANGPTL: angiopoietin-like protein; ANGPTL4: angiopoietin-like protein 4; ATP: adenosine triphosphate; ATGL: adipose triglyceride lipase; CD36/FAT: fatty acid translocase; CHD: coronary heart disease; CI: confidence interval; CL: cardiolipin; COX: cyclooxygenase; CPT: carnitine palmitoyltransferase; CPT1: carnitine palmitoyltransferase 1; CPT1A: carnitine palmitoyltransferase 1A; CPT1B: carnitine palmitoyltransferase 1B; CPT2: carnitine palmitoyltransferase 2; CRP: C-reactive protein; CVD: cardiovascular disease; CYP: cytochrome P450; CYP2J2: cytochrome P450 2J2; DAGs: diacylglycerols; DASH: Dietary Approaches to Stop Hypertension; DCM: dilated cardiomyopathy; DHA: docosahexaenoic acid, 22:6*n*-3; DRP1: dynamin-related protein 1; EFA: essential fatty acid; EETs: epoxyeicosatrienoic acids; ELOVL5/6: elongation of very long-chain fatty acids protein 5 and 6; EPA: eicosapentaenoic acid, 20:5*n*-3; ER: endoplasmic reticulum; ERK1/2: extracellular signal-regulated kinase 1/2; ETC: electron transport chain; EVOO: extra-virgin olive oil; FABP: fatty acid-binding protein; FABPpm: plasma membrane fatty acid-binding protein; FABPs: fatty acid-binding proteins; FACS: fatty acyl-CoA synthase; FADS1/2: fatty acid desaturase 1 and 2; FAT/CD36: fatty acid translocase; FATP: fatty acid transport protein; FATP1: fatty acid transport protein 1; FATPs: fatty acid transport proteins; FFAR: free fatty acid receptor; FFAR1 (GPR40): free fatty acid receptor 1; FFAR2 (GPR43): free fatty acid receptor 2; FFAR3 (GPR41): free fatty acid receptor 3; FFAR4 (GPR120): free fatty acid receptor 4; FFARs: free fatty acid receptors; FOXO1: forkhead box protein O1; FOXO3a: forkhead box protein O3a; GLUT1: glucose transporter 1; GPCR: G protein-coupled receptor; GPR120/FFAR4: free fatty acid receptor 4; HDL: high-density lipoprotein; HDAC: histone deacetylase; HETEs: hydroxyeicosatetraenoic acids; HFpEF: heart failure with preserved ejection fraction; HFrEF: heart failure with reduced ejection fraction; HR: hazard ratio; hs-CRP: high-sensitivity C-reactive protein; HSL: hormone-sensitive lipase; IF: intermittent fasting; IHD: ischemic heart disease; IKK: IκB kinase; IKur: ultra-rapid delayed rectifier potassium current; IL-1β: interleukin-1β; IL-6: interleukin-6; KD: ketogenic diet; KLF: Kruppel-like factor; KLF4: Kruppel-like factor 4; KLF5: Kruppel-like factor 5; KLF15: Kruppel-like factor 15; LA: linoleic acid, 18:2*n*-6; LCFA: long-chain fatty acid; LCFAs: long-chain fatty acids; LDL: low-density lipoprotein; LOX: lipoxygenase; Lpcat3: lysophosphatidylcholine acyltransferase 3; LPL: lipoprotein lipase; LTB_4_: leukotriene B_4_; LTC_4_: leukotriene C_4_; MCAD: medium-chain acyl-CoA dehydrogenase; MCD: malonyl-CoA decarboxylase; MCP-1: monocyte chemoattractant protein-1; MFN1: mitofusin 1; MFN2: mitofusin 2; MI: myocardial infarction; miRNAs: microRNAs; mtROS: mitochondrial reactive oxygen species; MUFA: monounsaturated fatty acid; MUFAs: monounsaturated fatty acids; NAD^+^: nicotinamide adenine dinucleotide; Nav1.5: sodium channel 1.5; NCoR: nuclear receptor co-repressor; NEFA: non-esterified fatty acid; NEFAs: non-esterified fatty acids; NF-κB: nuclear factor kappa-B; NLRP3: NLR family pyrin domain containing 3; OA: oleic acid, 18:1*n*-9; OPA1: optic atrophy 1; p38MAPK: p38 mitogen-activated protein kinase; PDK4: pyruvate dehydrogenase kinase 4; PGC-1α: PPARγ coactivator-1α; PGE_2_: prostaglandin E_2_; PGE_3_: prostaglandin E_3_; PKCθ: protein kinase Cθ; PP2A: protein phosphatase 2A; PPAR: peroxisome proliferator-activated receptor; PPARα: peroxisome proliferator-activated receptor alpha; PPARγ: peroxisome proliferator-activated receptor gamma; PPARδ: peroxisome proliferator-activated receptor delta; PUFA: polyunsaturated fatty acid; RCT: randomized controlled trial; ROS: reactive oxygen species; RXR: retinoid X receptor; SCD1: stearoyl-CoA desaturase 1; SCFAs: short-chain fatty acids; SERCA2a: sarco/endoplasmic reticulum Ca^2+^-ATPase 2a; SFA: saturated fatty acid; SIRT: sirtuin; SIRT1: sirtuin 1; SIRT3: sirtuin 3; SMRT: silencing mediator of retinoid and thyroid hormone receptors; SPMs: specialized pro-resolving mediators; SREBP: sterol regulatory element-binding protein; SREBP-1c: sterol regulatory element-binding protein 1c; SREBP-2: sterol regulatory element-binding protein 2; STAT3: signal transducer and activator of transcription 3; TAG: triglyceride; TFA: trans fatty acid; TG: triglyceride; TGF-β: transforming growth factor-beta; TLR: Toll-like receptor; TLR4: Toll-like receptor 4; TMAO: trimethylamine N-oxide; TNFα: tumor necrosis factor-alpha; TORC2: target of rapamycin complex 2; TRE: time-restricted eating; TXA_2_: thromboxane A_2_; TXB_2_: thromboxane B_2_; TXB_3_: thromboxane B_3_; UPR: unfolded protein response; VLDL: very low-density lipoprotein; VLSFA: very long-chain saturated fatty acid; VLSFAs: very long-chain saturated fatty acids; 15d-PGJ_2_: 15-deoxy-Δ12,14-prostaglandin J_2_.
